# Spectrum of Causative Pathogens and Resistance Rates to Antibacterial Agents in Bacterial Prostatitis

**DOI:** 10.3390/diagnostics11081333

**Published:** 2021-07-25

**Authors:** Alberto Trinchieri, Khalid Mohammed Abdelrahman, Kamran Hassan Bhatti, Jibril O. Bello, Krishanu Das, Ognyan Gatsev, Ivanka Gergova, Vittorio Magri, Nikos Mourmouras, Panagiotis Mourmouris, Soni Murdeshwar, Gianpaolo Perletti, Iliya Saltirov, Idrissa Sissoko, Konstantinos Stamatiou, Noor Buchholz

**Affiliations:** 1 School of Urology, University of Milan, 20100 Milan, Italy; 2 U Merge Ltd., London & Dubai, London 14561, UK; kamibhatti92@gmail.com (K.H.B.); jabarng@yahoo.com (J.O.B.); krishanudmd@gmail.com (K.D.); saltirov@vma.bg (I.S.); idrississoko@gmail.com (I.S.); scientific-office@u-merge.com (N.B.); 3 Urology Section, HMC Alkhor, Alkhor 3050, Qatar; krehman@hamad.qa; 4 Urology Unit, Department of Surgery, University of Ilorin Teaching Hospital, Ilorin 40001, Nigeria; 5 Bahrain Specialist Hospital, Juffair 10588, Bahrain; bshinfo@bsh.com.bh; 6 Clinic of Endourology and SWL, Military Medical Academy, 1606 Sofia, Bulgaria; gatsev@gmail.com (O.G.); gergova.ivana7@gmail.com (I.G.); 7 Urology Secondary Care Clinic, ASST-Nord, 20132 Milan, Italy; vittorio.magri@virgilio.it; 8 Department of Urology, Tzaneio Hospital, 18536 Piraeus, Greece; ourologiki@tzaneio.gr (N.M.); stamatiouk@gmail.com (K.S.); 9 Division of Urology II, National and Kapodistrian University of Athens, 10679 Athens, Greece; thodoros13@yahoo.com; 10 Department of Biotechnology and Life Sciences, Section of Medical and Surgical Sciences, University of Insubria, 21100 Varese, Italy; gianpaolo.perletti@uninsubria.it; 11 Faculty of Medicine and Medical Sciences, Ghent University, 9000 Ghent, Belgium; 12 Urology Department, Kati University Hospital, Kati 25091, Mali

**Keywords:** bacterial prostatitis, *Escherichia coli*, *Enterobacteriacae*, Gram-positive pathogens, antibiotic, resistance

## Abstract

Objective: To evaluate spectrum and resistance rates to antibacterial agents in causative pathogens of bacterial prostatitis in patients from Southern Europe, the Middle East, and Africa. Materials: 1027 isolates from cultures of urine or expressed prostatic secretion, post-massage urine or seminal fluid, or urethral samples were considered. Results: *Escherichia coli* (32%) and *Enterococcus* spp. (21%) were the most common isolates. Other Gram-negative, Gram-positive, and atypical pathogens accounted for 22%, 20%, and 5%, respectively. Resistance was <15% for piperacillin/tazobactam and carbapenems (both Gram-negative and -positive pathogens); <5% for glycopeptides against Gram-positive; 7%, 14%, and 20% for aminoglycosides, fosfomycin, and macrolides against Gram-negative pathogens, respectively; 10% for amoxicillin/clavulanate against Gram-positive pathogens; <20% for cephalosporins and fluoroquinolones against to Gram-negative pathogens (higher against Gram-positive pathogens); none for macrolides against atypical pathogens, but 20% and 27% for fluoroquinolones and tetracyclines. In West Africa, the resistance rates were generally higher, although the highest rates for ampicillin, cephalosporins, and fluoroquinolones were observed in the Gulf area. Lower rates were observed in Southeastern Europe. Conclusions: Resistance to antibiotics is a health problem requiring local health authorities to combat this phenomenon. Knowledge of the spectrum of pathogens and antibiotic resistance rates is crucial to assess local guidelines for the treatment of prostatitis.

## 1. Introduction

Infection is microbiologically proven in approximately 10% of patients presenting with prostatitis-like symptoms [1]. Acute prostatitis is characterized by urinary tract symptoms, pain, fever, malaise, and general symptoms. Sometimes, patients present with features of sepsis requiring hospitalization [2]. Mid-stream urine culture yields a diagnosis in most cases. In cases requiring hospitalization, blood cultures can additionally help. *Escherichia coli* is the most frequent pathogen in acute prostatitis. Other *Enterobacteriacae* may be causative, while *Pseudomonas aeruginosa* is less frequent, usually secondary to recent urological instrumentations. The rate of Gram-positive pathogens is approximately 5% [2].

Chronic bacterial prostatitis can present with varied clinical scenarios, including urinary symptoms, perineal, pelvic and genital pain, and sexual symptoms. Mid-stream urine cultures are positive in only a few cases. Other sources utilized for diagnosis include cultures of samples obtained through the four- or two-glass test [3], and culture of seminal fluid [4]. *E. coli* and other *Enterobacteriacae* are the most common pathogens isolated in chronic prostatitis [5], although recently, Gram-positive pathogens, particularly *Enterococci*, are increasingly reported [6]. The role of intracellular pathogens or “atypicals” (*Chlamydia trachomatis, Ureaplasma* spp., and *Mycoplasma* spp.) is still debated [7]. Acute prostatitis patients require high-dose parenteral antibiotics to be started prior to identifying the culprit microbe due to the clinical severity and potential risk of sepsis. During acute inflammation, the permeability of cell membranes is enhanced, and penetration of any antibiotic is facilitated [8]. Parenteral administration of high doses of broad-spectrum penicillins, third-generation cephalosporines, or fluoroquinolones is recommended in the acute phase, followed by an oral regimen with a susceptible antibiotic for at least four weeks [8].

Chronic prostatitis therapy requires the administration of drugs with high penetration in the prostatic tissue, which must be administered for long periods, preferably via the oral route, and must have good tolerability [1,8,9].

Drugs must be lipid-soluble, have favorable size and shape, and result in minimal serum protein binding to favor diffusion across biological membranes. In recent years, basic drugs such as trimethoprim, macrolides, and tetracyclines have been preferred, although fluoroquinolones (amphoteric drugs with two ionizing groups, positively and negatively charged) are now more often used [1].

Fluoroquinolones still remain the best available drug for the treatment of chronic bacterial prostatitis, although resistance rates to these antibiotics have increased in recent years. Recently, doubts have been raised about the tolerability of these drugs, and empirical use for prostatitis has been discouraged [10,11].

The effectiveness of fluoroquinolones can be enhanced by combining with macrolides [12], which, in addition to broadening the antibacterial efficacy spectrum, have other beneficial effects such as immunomodulatory activities and the prevention of biofilm formation [13]. On the contrary, macrolides can increase the toxicity of fluoroquinolones, because both drug classes can cause elongation of the QT interval of the electrocardiogram; thus, this combination should be used with caution.

Fosfomycin is another possible alternative to fluoroquinolones for the treatment of prostatitis [14,15,16].

Nitrofurantoin retains a favorable resistance profile, but its pharmacokinetic characteristics are not ideal for the treatment of prostate infections [17].

Glycopeptides could be an option for the treatment of Gram-positive pathogens, but these agents are not orally absorbed, and we have limited knowledge about their use in prostatitis—although, in selected cases of severe, multi-resistant Gram-positive prostatic infections, the efficacy of glycopeptides has been reported [18].

The effectiveness of the antibiotic treatment of bacterial prostatitis depends on the rates of microbial resistance to antibiotics commonly used to treat prostatic infections, which may change in different geographical areas as a result of environmental and sociocultural characteristics, and how infections are diagnosed and treated and the local use of antibiotics in people and animals.

The aim of this study was to assess the microbial spectrum in bacterial prostatitis cases in selected centers in Southeastern Europe, Africa, and the Gulf area, as well as their resistance pattern to commonly used antimicrobials.

## 2. Materials and Methods

A study was initiated by U-merge, an association that unites urologists around the world. Eight centers from seven countries, namely, Bulgaria, Greece (two centers), Italy, Qatar, Bahrain, Mali, and Nigeria, participated in this study. The results of microbiological investigations of patients with prostatitis-like symptoms were studied retrospectively. Age and type of clinical presentation (acute or chronic bacterial prostatitis) were recorded. Cultures of urine, prostatic secretions expressed by prostate massage (EPS), urine evacuated after prostate massage (VB3), seminal fluid and urethral specimens (urethral secretion, urethral swabs, and first void urine) were analyzed. Isolates were identified at the laboratory of each participating center using conventional methods according to local guidelines. Undiluted urine samples were cultured in blood and on MacConkey agar plates. Identification of traditional pathogens was performed by conventional methods or the Vitek-2 Compact (bioMerieux, Marcy l’Etoile, France) system. Isolates were tested for antimicrobial susceptibility using custom broth microdilution panels or disk diffusion. Minimum inhibitory concentration (MIC) values were evaluated according to guidelines of the Clinical and Laboratory Standards Institute (CLSI) (in most extra-European countries) or the European Committee for Antimicrobial Susceptibility Testing (EUCAST) (in European countries, plus Mali). The local interpretations of antimicrobial susceptibility testing, reported as S, I, or R, were considered for the analyses, as quantitative data (i.e., disk diffusion zone diameters or MIC values) were not provided by the participating laboratories. Different antimicrobial agents were included on the panels at each laboratory, according to the local guidelines, with appropriate dilution ranges. The identification and semi-quantitative assay for *M. hominis* and *U. urealyticum* were performed using the Mycoplasma IST 2 kit (bioMerieux). *C. trachomatis* was detected by direct immunofluorescence (monoclonal antibodies against lipopolysaccharide membrane, Kallestad). For each sample, the isolated pathogen, the source, and the antibiotic resistance pattern exhibited in the antibiogram were recorded. Each class of antibiotics was considered inactive on a specific bacterial strain only if it demonstrated resistance to all of the tested antibiotics of that class (i.e., any fluoroquinolone). Strains demonstrating intermediate susceptibility were not considered resistant. Resistance to a combination of quinolones–macrolides or amoxicillin/clavulanate-aminoglycosides was also assessed. The combination was classified as “resistant” if the pathogen was resistant to both agents.

### Statistical Analysis

The mean ages of patients in different countries were calculated, and any difference was analyzed with an ANOVA test. A *p*-value of <0.05 was considered as statistically significant. Post-hoc analysis was carried out with the Bonferroni test. Contingency tables were built (i) to compare the frequency of pathogens in different countries, (ii) to compare their antibiotic resistance rates, and (iii) to estimate how often different antibiotics were tested in the countries and for pathogens classified as Gram-negative, Gram-positive, and atypical pathogens. Differences between proportions were assessed with the chi-squared test or with a two-tailed *Z*-test for independent proportions.

## 3. Results

A total of 984 patients with acute (*N* = 60) and chronic (*N* = 924) bacterial prostatitis were considered. Patients were diagnosed in Bahrain (*N* = 50), Bulgaria (*N* = 202), Greece (*N* = 184), Italy (*N* = 409), Mali (*N* = 36), Nigeria (*N* = 30), and Qatar (*N* = 73). The average age was 46 ± 13 years. The mean ages were higher in Qatar (64 ± 11 years) than in Bahrain (43 ± 8 years), Bulgaria (44 ± 11 years), Greece (46 ± 12 years), Italy (45 ± 13 years), Mali (45 ± 14 years), and Nigeria (44 ± 10 years). The prevalence of acute prostatitis was higher in Mali (47%), Qatar (29%), Nigeria (27%), and Bahrain (14%), while a lower rate was observed in Greece (4%). No cases of acute prostatitis were reported from Bulgaria and Italy. The average age of patients with acute prostatitis was not significantly higher than those with chronic prostatitis (48 ± 15 vs. 46 ± 13, *p* > 0.005, ANOVA). Overall, 1027 pathogens were isolated: A single pathogen in 942 patients, two pathogens in 41 patients, and three pathogens in one patient.

### 3.1. Source of Samples for Microbiological Diagnosis

**Figure 1:**
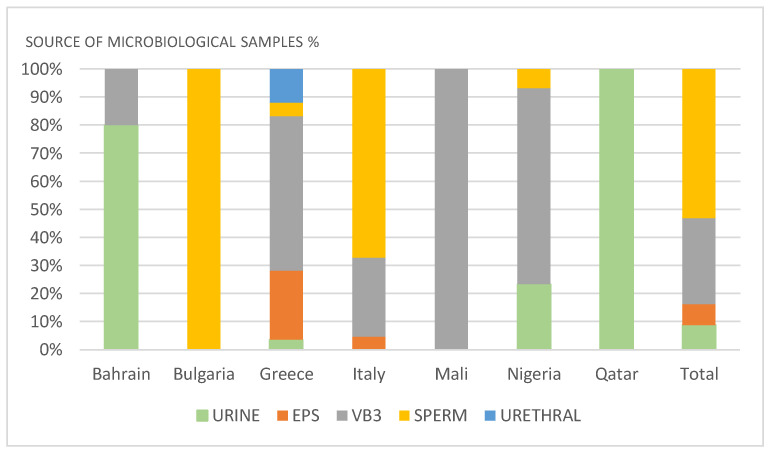
Source of samples for microbiological diagnosis.

Bacterial prostatitis was diagnosed by midstream urine culture in 88 cases, by EPS culture in 29 cases, by VB3 culture in 307 cases, by EPS + VB3 culture in 46 cases, by sperm culture in 530 cases, and by culture of urethral samples in 27 cases. In Qatar, pathogens were isolated from urinary samples, in Bulgaria from seminal fluid, and in Mali VB3. In Bahrain, pathogens were isolated from seminal fluid in 80% and VB3 in 20% of samples; in Nigeria, from VB3 in 70%, urine in 23%, and seminal fluid in 7% of samples; in Greece, from VB3 in 68%, urine in 4%, EPS or EPS + VB3 in 30%, seminal fluid in 6%, and urethral samples in 15% of samples; in Italy, from seminal fluid in 67%, VB3 in 28%, and EPS in 5% of samples (Figure 1).

### 3.2. Acute and Chronic Prostatitis

**Figure 2:**
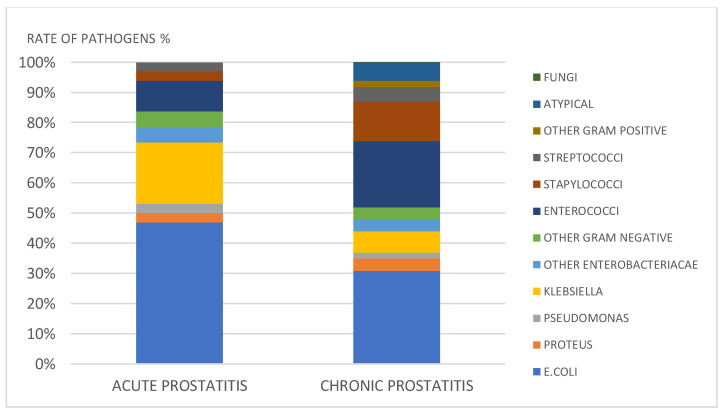
Pathogens in acute and chronic prostatitis.

The most frequent isolate was *E. coli*, detected in 46% of acute and 31% of chronic cases (*p* = 0.029). *Klebsiella* spp. was more frequent in acute prostatitis than in chronic prostatitis (20% vs. 7% of cases, *p* = 0.0071). The rates of *Proteus* spp. (3% vs. 4%), *P. aeruginosa* (3% vs. 2%), other *Enterobacteriacae* (5% vs. 4%), and other Gram-negative bacteria (5% vs. 4%) were similar in the two groups (*p* > 0.05). *Enterococci* (10% vs. 22%, respectively, *p* = 0.020), *Staphylococci* (3% vs. 13%, *p* = 0.0002), *Streptococci* (3% vs. 5% *p* > 0.05), and other Gram-positive pathogens (0% vs. 2% *p* > 0.05) were less frequent in acute compared to chronic prostatitis. Atypical pathogens (6%) and fungi (0.1%) were isolated only from chronic prostatitis specimens (Figure 2).

### 3.3. Microbiological Samples

In midstream urine samples, *E. coli* and other Gram-negative and Gram-positive pathogens accounted for 44%, 43%, and 14%. In VB3 samples, *E. coli*, other Gram-negative and Gram-positive pathogens, atypical pathogens, and fungi were found in 40%, 19%, 37%, 3%, and 0.3% of cases, respectively. In EPS, LS, and urethral samples, *E*. *coli* accounted for 25%, 26%, and 22% of the cases; other Gram-negative pathogens for 9%, 24%, and 11% of cases; Gram-positive pathogens for 62%, 44%, and 33% of cases; and atypical pathogens for 1%, 7%, and 22% of cases, respectively.

### 3.4. Spectrum of Pathogens in Different Countries

**Figure 3:**
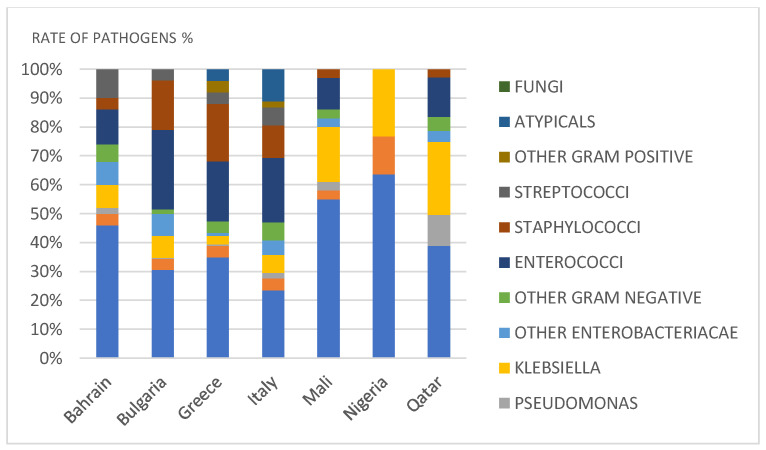
Spectrum of pathogens in different countries.

*E. coli* was common in Nigeria (63%), Mali (55%), Bahrain (46%), Qatar (40%), Greece (35%), Bulgaria (32%), and Italy (23%). *Proteus* spp. were less frequent (3–4%), except in Nigeria (13%). *P. aeruginosa* (0–2%) was also less prevalent, except in Qatar (11%). *Klebsiella* spp. ranged between 3% and 8% in Bahrain, Bulgaria, Greece, and Italy, but higher rates were observed in Qatar (26%), Nigeria (23%), and Mali (19%). Rates of other *Enterobacteriacae* (1–8%) and other Gram-negative pathogens (1.5–6%) were low in all countries. Gram-positive bacteria were frequently isolated in Greece, Bulgaria, and Italy (41–51%) with *Enterococci* being the most frequent isolate. Lower rates of Gram-positive pathogens were observed in Qatar (17%), Bahrein (27%), Nigeria (0%), and Mali (14%). Atypical pathogens were observed in Greece (4%) and Italy (11%) (Figure 3).

### 3.5. Antibacterial Susceptibility Testing in Different Countries

The most frequently tested antibiotics were amoxicillin/clavulanate (57–100%), cephalosporins (60–100%), fluoroquinolones (79–100%), aminoglycosides (41–100%), carbapenems (37–74%), and piperacillin–tazobactam (21–97%; not tested in Nigeria). Some antibiotics were not tested, or only tested in a few cases in some countries: In Bahrain, ampicillin in 4% and tetracyclines and fosfomycin in none; in Qatar, glycopeptides in 9% of cases and macrolides, tetracyclines, and fosfomycin in none; in Mali, trimethoprim sulfamethoxazole (TMP/SMX) in 3% and glycopeptides in none; in Nigeria, piperacillin/tazobactam, glycopeptides, macrolides, tetracyclines, and fosfomycin were never tested.

### 3.6. Antibiotic Resistance Rates in Different Countries

**Figure 4:**
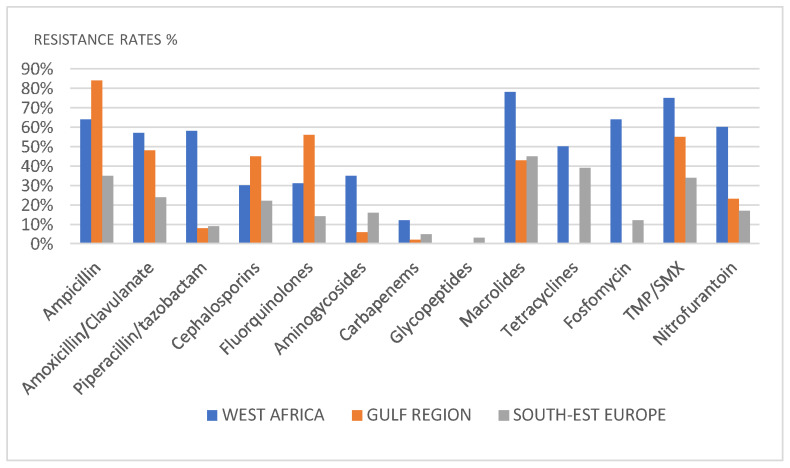
Resistance rates by geographical area.

The antibiotic resistance rates in different countries are shown in Figure 4.

### 3.7. Antibiotic Susceptibility Testing in Different Pathogens

Antibiotics tested in both Gram-negative and Gram-positive pathogens were ampicillin (69% and 82%), amoxicillin/clavulanate (87% and 67%), fluoroquinolones (96% and 80%), and aminoglycosides (79% and 78%). Gram-negative pathogens were tested for piperacillin–tazobactam (64%), cephalosporins (98%), carbapenems (84%), fosfomycin (50%), TMP-SMX (63%), and nitrofurantoin (35%). Less than half of Gram-negative pathogens were tested for glycopeptides (10%), macrolides (8%), and tetracyclines (14%). Gram-positive pathogens were tested for glycopeptides (88%), macrolides (69%), cephalosporins (54%), and carbapenems (50%). Less than half of Gram-positive isolates were tested for piperacillin/tazobactam (46%), tetracyclines (44%), fosfomycin (24%), TMP-SMX (41%), and nitrofurantoin (33%). Atypical pathogens were tested for macrolides, tetracyclines, and quinolones.

### 3.8. Resistance to Antibacterial Agents in Different Pathogens

The antibiotic resistance rates of pathogens are reported in Table 1 and Table 2.

### 3.9. Resistance to Antibiotics Used in Combined Antibiotic Regimens

Only 13% of pathogens were simultaneously resistant to quinolones and macrolides, while resistance was ascertained to one of them in 37%.

In 4% of pathogens, there was simultaneous resistance to amoxicillin/clavulanate and aminoglycosides, while resistance to one of them was present in 38%.

## 4. Discussion

In general, our data confirmed that *E. coli* is the most common isolate in prostate infections, although the frequency of *E. coli* was higher in acute prostatitis (46%) than in chronic prostatitis (31%).

In our series, Gram-positive bacteria were isolated in 17% of acute prostatitis, but in 42% of chronic prostatitis, in congruence with recent reports of increasing incidence of these pathogens, particularly *Enterococci*, in chronic prostatitis cases [19,20].

The frequency of *E. coli* was higher in West African countries (55–63%) and in the Gulf countries (40–46%) than in European countries (23–35%) due to the different acute to chronic infections ratio. In fact, acute infections were more frequently reported in West Africa (27–47%) and the Gulf area (14–29%) than in Southeastern Europe. Conversely, Gram-positive bacteria were more frequently isolated in Greece, Bulgaria, and Italy (41–51%) compared to Qatar (17%), Bahrain (27%), Nigeria (0%), and Mali (14%). Atypical pathogens were researched and observed only in Greece (4%) and Italy (11%).

Cumulative resistance rates to some antibiotics, such as carbapenems and piperacillin/tazobactam, are still low in both Gram-negative and -positive pathogens. The resistance rates of Gram-positive pathogens also remained low for glycopeptides and amoxicillin/clavulanate. Conversely, the resistance rates to cephalosporines and fluoroquinolones in Gram-negative pathogens were approximately 20%, but higher in Gram-positive pathogens (43% and 29%, respectively). Finally, the resistance rates of atypical pathogens were 20% and 27% for fluoroquinolones and tetracyclines, while no resistance was observed for macrolides.

The rates of antibiotic resistance were different in different geographical areas. Three different geographical scenarios with similar environmental and sociocultural characteristics and healthcare system organization were observed (West Africa, Persian Gulf, and Southeastern Europe).

### 4.1. West Africa

In West African countries, citizens are eligible for subsidized public healthcare—some have private health insurance, while some are not insured at all.

The study cohort from West Africa mainly comprised patients with acute prostatitis and severe symptoms seeking medical attention. Chronic infections seem to be unattended or self-managed. The panel of antibiotics utilized and tested for susceptibility in these countries is limited. Additionally, resistance rates for antibiotics are much higher than in other countries.

Our findings confirm those of a systematic review of studies focused on urinary tract infections demonstrating high rates of antibiotic resistance in West Africa [21]. Among *E. coli* isolates, resistance was reported in 74.5–81% for ampicillin, 38.8–52.5% for amoxicillin/clavulanate, 26–47.7% for ceftazidime, 9.3–37.7% for gentamicin, 11.7–24% for ciprofloxacin, and 60.4–81% for TMP/SMX. Ouedraogo et al. [22] attributed this increased antibiotic resistance to inadequate diagnostic tools and training of prescribers, self-medication, and an uncontrolled drug sector (antibiotics sold over-the-counter, improperly stored, counterfeit, and/or expired).

### 4.2. Persian Gulf

Persian Gulf countries have health systems of high standard freely accessible for citizens, but not expatriates, who should rely on private health insurance or pay out the expenses for health services and medications themselves. The microbiological epidemiology in this area was somewhat similar to that in West Africa, although the panel of antibiotics tested was larger and the resistance rates lower. Increasing antibiotic resistance has been previously reported in the Gulf area [23]. In the United Arab Emirates [24], from 1999–2002 to 2008, there was an increase in *E. coli* resistance to ceftazidime from 9% to 22.7% and to ciprofloxacin from 19.9% to 34.8%. The resistance rates for TMP/SMX and ampicillin were already high in 1999–2002, while the resistance rates for imipenem (0–0.1%), gentamicin (11.1–17.7%), and piperacillin/tazobactam (2.1–4.9%) remained low. Self-medication seems to be one of the main causes of this increase in resistance. A study in Abu Dhabi [25] confirmed that antibiotic self-medication is relatively frequent, with 46% of surveyed participants stating that they intentionally used antibiotics as self-medication without medical consultation.

### 4.3. Southeastern Europe

Southeastern European countries have universal government-funded or mixed public–private health systems covering diagnostic services, as well as medications.

Data from Southeastern Europe are mostly related to chronic prostatitis, which requires more complex and expensive diagnostics and prolonged therapies. Access to diagnostics and the treatment of chronic diseases at no direct cost to the patient could explain the greater number of these cases seeking medical attention in European countries with universal health systems. A larger number of antibiotics were tested with a high resistance pattern, although the resistance rates were lower than those observed in other countries of the survey.

Resistance rates <20% were assessed for carbapenems, piperacillin/tazobactam, aminoglycosides (excluding Bulgaria), cephalosporins (excluding Bulgaria), fluoroquinolones, and fosfomycin (excluding Italy).

Other studies [26,27] have reported higher antibiotic resistance rates in Southeastern European countries that could be attributed to the over-usage of antibiotics for trivial scenarios.

### 4.4. Options for the Antibiotic Treatment of Bacterial Prostatitis in Different Geographical Areas

International guidelines recommend the empirical treatment of acute bacterial prostatitis with parenteral administration of high doses of bactericidal antimicrobials, such as broad-spectrum penicillins, a third-generation cephalosporin, or fluoroquinolones, that may be combined with an aminoglycoside [28]. Carbapenems should be reserved for the most severe cases and administered in an inpatient setting.

According to our data, this option seems to be feasible in Southeastern Europe or the Gulf area, where resistance to piperacillin–tazobactam is less than 10% and to the association between amoxicillin/clavulanate with an aminoglycoside is <5%.

The scenario is more critical in West Africa, where the resistance rate to piperacillin/tazobactam in Mali has been reported to be 58%, and the drug was never tested in Nigeria. The resistance to the amoxicillin/clavulanate and aminoglycoside combination is <10% in Mali, but very high in Nigeria, where the resistance rates to amoxicillin/clavulanate are 100% and to aminoglycosides are 60%.

The guidelines still recommend fluoroquinolones as first-line agents in the empirical treatment of chronic bacterial prostatitis, despite the increasing resistance rates of uropathogens to fluoroquinolones.

According to our data, the resistance rates in Southeastern Europe for fluoroquinolones are 15%, while much higher rates have been observed in the Gulf area and in West Africa. On the contrary, TMP-SMX, macrolides, and tetracyclines showed resistance rates > 20% in most countries.

The association between fluoroquinolones and macrolides may represent an alternative option to be used with caution due to the risk of cardiotoxicity. The microbiological data looks promising in most countries, although susceptibility to macrolides was not tested in Qatar or Nigeria.

Fosfomycin is another possible alternative to fluoroquinolones for the treatment of prostatitis. In our study, the resistance rates to fosfomycin were less than 10% in Greece and Bulgaria and 23% in Italy. A high resistance rate was observed in Mali, while susceptibility to the antibiotic was not tested in the Gulf area or Nigeria.

### 4.5. Limitations

A limitation of our study is the possible discrepancy resulting from microbiological investigations in different laboratories using different methodologies and guidelines for clinical breakpoints of antibiotic susceptibility. As a result, the interpretation of antimicrobial susceptibility testing results may vary, at least for resistance estimates close to the breakpoints.

On the contrary, the main objective of our study was not a comparison between the resistance rates in different countries, which requires a more rigorous methodology with standardization or centralization of the investigations, but rather a description of the epidemiological aspects based on the microbiological diagnostics available in real life. The data obtained are certainly preliminary, but can be used to plan subsequent investigations that become mandatory due the globalization of health problems.

## 5. Conclusions

The results of this survey provide us some information about the microbiology and resistance rates of pathogens of prostatic infections, although caution should be exercised when comparing resistance rates across countries, because of differences in sample collections and testing methods. Taking into account these limitations, data from this study may be useful to clinicians to formulate their policies for the empirical treatment of prostatitis; to clinicians in planning treatment of migrant patients or patients resident in other countries for long time; to local health authorities in creating appropriate guidelines based on the microbiological epidemiology of their countries and to put in place medical and veterinary policies to reduce the emergence of further resistance, particularly for those few drugs that still retain high levels of efficacy; to international health organizations in building antibiotic stewardship programs that will limit antibiotic multi-resistant strains.

## Figures and Tables

**Table 1:** Resistance rates of Gram-negative pathogens.

	*E. coli*	*Proteus*	*Pseudom*	*Klebs*	Other Gram-Neg	Total Gram-Neg
Ampicillin	112/238 47%	17/24 71%	6/6 100%	60/67 90%	43/52 100%	238/387 61%
Amox/Clav	76/290 26%	10/34 29%	9/9 100%	44/78 56%	51/75 76%	190/486 39%
Pip/Taz	27/220 12%	3/27 11%	1/17 6%	6/51 12%	4/43 7%	41/358 11%
Cephalo	62/321 19%	5/40 12%	1/22 5%	26/84 31%	8/79 18%	101/546 18%
Quinolones	66/321 20%	5/40 12%	2/22 9%	24/78 31%	9/76 14%	106/537 18%
Aminogly	21/300 7%	2/39 5%	2/22 9%	8/75 11%	2/3 0%	36/499 7%
Carbapen	10/289 3%	0/38 0%	1/11 9%	3/75 4%	1/35 3%	17/469 4%
Glycopep	4/45 9%	0/5 0%	0 0%	0/3 0%	0/1 0%	4/55 7%
Macrolides	7/34 20%	0/2 0%	0 0%	0/2 0%	0 0%	9/44 20%
Tetracycl	9/50 18%	3/5 60%	2/2 100%	3/7 43%	2/3 67%	21/77 27%
Fosfomycin	21/195 11%	4/18 22%	1/1 100%	7/37 19%	4/19 21%	40/277 14%
TMP/SMX	60/224 27%	9/24 37%	4/4 100%	22/54 41%	5/22 23%	103/354 29%
Nitrofur	23/148 15%	3/12 25%	3/3 100%	8/16 50%	2/7 29%	45/193 23%

**Table 2:** Resistance rates of Gram-positive and atypical pathogens.

	*Entero*	*Staphylo*	*Strepto*	Other Gram-Pos	Total Gram-Pos	Atypicals
Ampicillin	24/207 12%	32/90 35%	1/37 3%	1/8 12%	58/342 17%	-
Amox/Clav	14/152 9%	13/93 14%	0/28 0%	0/7 0%	27/280 10%	-
Pip/Taz	9/93 10%	13/79 16%	0/16 0%	0/2 0%	22/190 12%	-
Cephalo	81/87 93%	14/89 16%	1/41 2%	1/6 17%	97/223 43%	-
Quinolones	38/182 21%	23/10 21%	1/27 4%	6/15 40%	68/234 29%	9/44 20%
Aminogly	73/169 43%	20/123 16%	12/27 44%	2/10 20%	107/329 32%	-
Carbapen	6/109 6%	12/81 15%	0/17 0%	0/2 0%	18/209 9%	-
Glycopep	1/196 0.5%	6/119 5%	0/40 0%	0/11 0%	7/366 2%	-
Macrolides	102/133 77%	49/117 42%	8/31 26%	1/5 20%	160/286 56%	0/43 0%
Tetracycl	41/58 71%	30/104 29%	4/8 50%	5/13 38%	80/183 44%	3/11 27%
Fosfomycin	8/32 25%	12/57 21%	0/8 0%	0/1 0%	18/98 18%	-
TMP/SMX	72/75 96%	23/75 31%	1/11 9%	4/9 44%	100/170 59%	-
Nitrofur	14/75 19%	10/47 21%	0/8 0%	1/6 17%	25/136 18%	-

## Data Availability

The datasets analyzed during the current study are available from U-merge on reasonable request.
